# JC Virus Small t Antigen Binds Phosphatase PP2A and Rb Family Proteins and Is Required for Efficient Viral DNA Replication Activity

**DOI:** 10.1371/journal.pone.0010606

**Published:** 2010-05-12

**Authors:** Brigitte Bollag, Catherine A. Hofstetter, Marta M. Reviriego-Mendoza, Richard J. Frisque

**Affiliations:** Department of Biochemistry and Molecular Biology, The Pennsylvania State University, University Park, Pennsylvania, United States of America; Yonsei University, Republic of Korea

## Abstract

**Background:**

The human polyomavirus, JC virus (JCV) produces five tumor proteins encoded by transcripts alternatively spliced from one precursor messenger RNA. Significant attention has been given to replication and transforming activities of JCV's large tumor antigen (TAg) and three T′ proteins, but little is known about small tumor antigen (tAg) functions. Amino-terminal sequences of tAg overlap with those of the other tumor proteins, but the carboxy half of tAg is unique. These latter sequences are the least conserved among the early coding regions of primate polyomaviruses.

**Methodology and Findings:**

We investigated the ability of wild type and mutant forms of JCV tAg to interact with cellular proteins involved in regulating cell proliferation and survival. The JCV P99A tAg is mutated at a conserved proline, which in the SV40 tAg is required for efficient interaction with protein phosphatase 2A (PP2A), and the C157A mutant tAg is altered at one of two newly recognized LxCxE motifs. Relative to wild type and C157A tAgs, P99A tAg interacts inefficiently with PP2A *in vivo*. Unlike SV40 tAg, JCV tAg binds to the Rb family of tumor suppressor proteins. Viral DNAs expressing mutant t proteins replicated less efficiently than did the intact JCV genome. A JCV construct incapable of expressing tAg was replication-incompetent, a defect not complemented in *trans* using a tAg-expressing vector.

**Conclusions:**

JCV tAg possesses unique properties among the polyomavirus small t proteins. It contributes significantly to viral DNA replication *in vivo*; a tAg null mutant failed to display detectable DNA replication activity, and a tAg substitution mutant, reduced in PP2A binding, was replication-defective. Our observation that JCV tAg binds Rb proteins, indicates all five JCV tumor proteins have the potential to influence cell cycle progression in infected and transformed cells. It remains unclear how these proteins coordinate their unique and overlapping functions.

## Introduction

JC virus (JCV) belongs to the *Polyomaviridae* family of small double-stranded DNA tumor viruses that includes four other human polyomaviruses: BKV, WUV, KIV and MCV. These five viruses are distributed globally among the human population with seroprevalence ranging from 39% to 82% among healthy adult blood donors [1]. Some studies have suggested that simian virus 40 (SV40), the prototype member of the primate polyomavirus subgroup, also circulates in humans as a consequence of exposure to virus present in early preparations of poliovirus vaccine. The JCV, BKV and SV40 genomes share a high degree of sequence homology (69–75%) and organization of the viral genes is nearly identical [2], but the viruses do exhibit distinct biological differences. For example, JCV exhibits restricted growth and oncogenic potential in cell culture, in part due to highly tissue-specific transcriptional signals and early regulatory proteins that appear to be less robust than those of SV40 [reviewed in 3]–[6]. A comparison of the primate polyomavirus genomes indicates that promoter-enhancer sequences have diverged to the greatest extent. JCV produces five early proteins, the large tumor antigen (TAg), small tumor antigen (tAg), and three T′ proteins, which share overlapping N-terminal sequences and exhibit common and unique replication and transforming functions [7], [8]. The SV40 genome encodes three early proteins, TAg, tAg, and 17KT that, like the JCV early regulatory proteins, are encoded by alternatively spliced early transcripts.

TAg is the major polyomavirus tumor protein. At least three distinct TAg domains contribute to oncogenic transformation. The J domain, named for functional and sequence similarities to cellular DnaJ co-chaperones, binds to the molecular chaperone Hsc70. This domain, in cooperation with a second motif, the LxCxE domain, regulates cell cycle progression, in part, by interacting with the Rb family of proteins, activating the intrinsic ATPase activity of Hsc70 and effecting the release of members of the E2F family of transcription factors from their Rb partners [reviewed in 9]. SV40 TAg also interacts with insulin receptor substrate 1 (IRS1) through its LxCxE domain, leading to activation of PI3 kinase (PI3K), which in turn up-regulates phosphorylation of Akt [10]. The third transformation domain of TAg is a C-terminal bipartite region that directly binds and inactivates the tumor suppressor protein p53 [11]. Binding of SV40 TAg to p53 promotes the recruitment of CBP/p300, which in turn influences TAg acetylation [12], [13] and stability, and impacts oncogenic transformation of NIH-3T3 cells [14]. JCV TAg has also been reported to interact with β-catenin, contributing to cellular transformation. β-catenin, a key member of the Wnt pathway, is stabilized and imported to the nucleus through a physical interaction with JCV TAg where it up-regulates expression of proteins involved in cell growth and proliferation [15].

Although the oncogenic mechanisms of the primate polyomavirus TAgs have received much attention, the essential activities of this multifunctional protein relate to its role in mediating viral DNA replication. Many of the TAg sequences required for initiation and elongation of replication reside in the unique C-terminal region of the protein [5]. However, N-terminal sequences shared with the other tumor proteins, including tAg and the TAg splice variants (17KT, T′ proteins), also influence viral DNA replication. For example, the J domain of TAg is required for efficient viral DNA replication [16], yet few data are available that address the replication functions of these same sequences in the other tumor proteins.

JCV tAg has only recently become a focus of study; however, a number of functions of the related SV40 tAg are known. SV40 tAg cooperates with TAg to enhance transformation when TAg levels are low or quiescent cells are being tested [reviewed in 17], [18]. SV40 tAg expression is dispensable for TAg-mediated transformation of dividing rodent cells, but is required for transformation of human cells [19]. In addition, tAg stimulates, but is not essential to, viral replication in permissive cells [20]. This stimulation likely results from the ability of tAg to alter the activity of cellular protein phosphatase 2A (PP2A), which in turn may lead to changes in the phosphorylation state of critical serine and threonine residues regulating TAg replication functions [reviewed in 20], [21]. SV40 tAg is also critical to the maintenance of high viral DNA copy numbers in persistent infections of human mesothelial cells; in the absence of tAg, cells are immortalized but not transformed, and only low copy numbers are maintained [22].

Unique sequences within the SV40 and murine polyomavirus tAgs are responsible for the binding of PP2A [23]–[25], a phosphatase that regulates numerous cell activities by dephosphorylating a variety of substrates. Making up as much as 1% of total cell protein [26], PP2A is a modular holoenzyme consisting of three subunits. A large number of regulatory subunit B isoforms directs functional specificity when joined to the core enzyme composed of the scaffolding subunit A and the catalytic subunit C [25], [26]. For example, pools of holoenzymes with specific B subunits are formed differentially during the course of the cell cycle, and the PP2A holoenzymes with cell-cycle-specific regulatory subunits orchestrate cell cycle progression [27]. Various viral proteins form complexes with the AC core [21], and some partially displace B subunits already complexed in the holoenzyme [24]. Through co-immunoprecipitation (co-IP) experiments, SV40 tAg was associated with the A and C subunits of PP2A [28], [29]. Addition of SV40 tAg to the AC core alters the subcellular localization of PP2A [30] as well as the phosphatase activity of the holoenzyme, and modulates protein kinase signaling pathways [21], [31], thus potentially influencing the cell cycle.

SV40 tAg facilitates the transformation of cells by activating phosphorylation of PI3K in a PP2A-dependent fashion, which in turn up regulates the activity of multiple signal transduction pathways [reviewed in 32]. The cellular kinase Akt, part of the PI3K-Akt pathway, contributes to enhanced activity of telomerase, a necessary but not sufficient step in the oncogenic progression of human cells [33]. Akt has two phosphorylation sites: T308, in the activation loop of the kinase, and S473, at the carboxyl terminus. Depending on cell growth conditions, phosphorylation of T308 alone or T308 and S473 together may be stimulated through the PI3K pathway following binding of PP2A by tAg [34]. TAg has also been shown to effect modification of both residues in Akt through its binding to IRS1 [10].

Here, we have examined the interaction of wild-type and mutant JCV tAgs with PP2A and members of the Rb family in cultured cells, and have investigated tAg functions that may influence JCV transforming and DNA replication potential.

## Materials and Methods

### Generation of Viral DNA Constructs

We have adopted a uniform naming scheme for the recombinant DNAs (rDNAs) constructed for this study. For clarity, plasmids previously described have been renamed according to this scheme, with the original name indicated in parentheses after the first mention of each construct in the text. Expression of TAg (T), tAg (t), or the three T' proteins (T′_135_, T′_136_, T′_165_) is indicated by a plus (+); lack of expression is denoted by a minus (-) sign. An amino acid substitution, for example the alteration of proline at residue 99 of tAg to alanine, is indicated by the single letter abbreviation of the wild type (P) and altered (A) amino acid with the residue number (99) identified between these letters. The viral source of the transcription signals effecting gene expression is noted at the start of each name. JR, SR and CMV denote the JCV, SV40 and cytomegalovirus regulatory regions, respectively. Therefore, the rDNA, SR:T^+^/P99At^+^/T′^+^, expresses the JCV wild type TAg and T′ proteins and the mutant P99A tAg under the control of the SV40 promoter/enhancer signals.

pCMV:T^+^/t^+^/T′^+^ (previously pCMV-JCV_E_; [8]) expresses all five JCV early proteins via the CMV early promoter in the pCR3 vector (Invitrogen). pCMV:T^+^/t^−^/T′^−^ (previously pCMV-ΔT′; [8]) expresses TAg only. pCMV:T^+^/t^−^/T′^+^ (previously pCMV-JCT; [8]) expresses TAg and the three T′ proteins, but not tAg.

pJR:T^+^/t^−^/T′^+^ was constructed by first digesting pCMV:T^+^/t^−^/T'^+^ with *Nco*I and *Pst*I to isolate the DNA fragment containing TAg cDNA coding sequences. This fragment was ligated to pJR:T^+^/t^+^/T′^+^ (previously pMRMMT; [35]) which had been digested with the same restriction enzymes to remove the corresponding sequence. To create a second construct expressing these same JCV proteins but under the control of SV40 transcription signals (pSR:T^+^/t^−^/T'^+^), the JCV regulatory region was removed from pJR:T^+^/t^−^/T′^+^ by *Nco*I cleavage and replaced with the SV40 regulatory sequences obtained by *Nco*I digestion of pSR:T^+^/t^+^/T'^+^ (previously pSRMMT; [35]). The expected structures of the pJR:T^+^/t^−^/T′^+^ and pSR:T^+^/t^−^/T'^+^ rDNAs were confirmed by DNA sequence analysis.

PCR was performed to generate a JCV tAg expression plasmid. An mRNA template from PHFG cells transfected with JCV DNA was amplified with primers tAg cDNA R (5′-GAG CTT ATG GAT TTA TTA GGC CTT GAT AGG TCT GCA TGG-3′), nt 4980–4942, and tAg cDNA F (5′-GCT ATC CAT AGG TTG GCA CCT TAA AGC TTT AGA TCC CTG TAG G-3′), nt 4412–4426, 4494–4517 with 4 non-JCV nt at the 5′ end. The product, a DNA fragment containing the tAg cDNA, was ligated to the linear TA cloning vector pCR2.1 (Invitrogen) and sequenced. The tAg cDNA contained one mutation, an artifact of the PCR amplification step, at nucleotide (nt) 4605. The resulting plasmid was digested with *Eco*NI and *Pfl*MI to isolate the tAg cDNA, which was ligated to pCMV:T^+^/t^−^/T′^+^ from which the corresponding sequence had been removed. The resulting mutant tAg expression vector, pCMV:T^−^/mut^+^/T'^−^, was subjected to PCR-mediated site-directed mutagenesis to correct the point mutation using primers WTtAgL (5′-GCA ATC AAA GCA ATA GCA ATC TAT CCA CAC AAG TGG GC -3′), and WTtAgE (5′-GCC CAC TTG TGT GGA TAG ATT GCT ATT GCT TTG ATT GC-3′), which span the coding sequence from nt 4628–4591. The tAg coding region of the PCR product was confirmed by DNA sequence analysis. The sequenced region, which contained the wild type tAg cDNA sequence, was isolated and ligated to the pCMV:T^−^/mut^+^/T'^−^ backbone with the corresponding region removed. This step ensured that any secondary mutations inadvertently introduced via PCR would not be present in the final construct. Next, the tAg cDNA region was isolated following *Bst*XI and *Pst*I digestion and ligated to pJR:T^+^/t^+^/T′^+^ with the corresponding sequence removed, to create pJR:T^−^/t^+^/T'^−^. The expected structure of the rDNA was confirmed by DNA sequence analysis.

pJR:T^+^/t^+^/T′^+^ and pSR:T^+^/t^+^/T′^+^ contain wild-type JCV coding sequences linked to either the JCV or SV40 regulatory region, respectively. Each plasmid was used as a template for site-directed mutagenesis to generate constructs encoding P99A or C157A mutant tAgs. Complementary primers P99AR (5′-CC CTT TAT TGC AAG GAA TGG **G**CT AAC TGT GCC ACT AAT CC-3′) and P99AF( 5′-GG ATT AGT GGC ACA GTT AG**C** CCA TTC CTT GCA ATA AAG GG-3′), which span the coding sequence from nt 4739–4700, or C157AF (5′-CCC AAG AAG CTC TTC ATT **C**CT GGG AGA AAG TTC TTG G-3′) and C157AR (5′-CCA AGA ACT TTC TCC CAG **G**AA TGA AGA GCT TCT TGG G -3′), which span the coding sequence from nt 4562–4526, were used. Altered nucleotides are in bold and underlined. The PCR products were sequenced, and the regions of interest were removed by BstXI and PflMI digestion. The fragments containing the point mutations were ligated to pJR:T^+^/t^+^/T′^+^ or pSR:T^+^/t^+^/T′^+^ backbone vectors to create pJR:T^+^/P99At^+^/T′^+^, pJR:T^+^/C157At^+^/T′^+^, pSR:T^+^/P99At^+^/T′^+^ and pSR:T^+^/C157At^+^/T′^+^. The structure of each construct was confirmed by DNA sequence analysis.

To generate pJR:T^−^/H42Qt^+^/T′^−^, PCR-mediated site-specific mutagenesis was performed on pSR:T^+^/t^+^/T′^+^ using primers H42Qf (5′-CCCCACCTTTATCAGG**T**TGGAGTTCTTTGC-3′) and H42Qr (5′-GCAAAGAACTCCA**A**CCTGATAAAGGTGGGG-3′), which span the coding sequence from nt 4901–4872. The region spanning the H42Q mutation in the cloned PCR amplicon was subjected to DNA sequence analysis, isolated by restriction enzyme digestion, and ligated to the pJR:T^−^/t^+^/T′^−^ backbone from which the corresponding fragment was removed via digestion. The rDNA structure was confirmed by DNA sequence analysis.

### Cell Culture

Primary human fetal glial (PHFG), Rat2, mouse embryo fibroblast (MEF) and 3T3 (3T3-Swiss albino; American Type Culture Collection) cells were cultured in Dulbecco's modified Eagle's medium (DMEM) supplemented with 10% (PHFG) or 5% (R2, MEF, 3T3 cells) fetal bovine serum (FBS), 100 U/ml penicillin and 0.1 mg/ml streptomycin. U-87MG cells were cultured in minimum essential Eagle's medium (MEM) with 10% FBS, 2% sodium pyruvate, 100 U/ml penicillin and 0.1 mg/ml streptomycin. All cells were incubated at 37°C in 10% CO_2_.

### Generation of Cell Lines

To create cell lines stably expressing all 5 JCV early proteins (T^+^/t^+^/T′^+^, T^+^/P99At^+^/T′^+^, T^+^/C157At^+^/T′^+^), or small tAg alone (T^−^/t^+^/T′^−^), 3T3 cells in 100 mm dishes were co-transfected with 5 µg of DNA encoding JCV proteins driven by SV40 promoter-enhancer signals, and 0.5 µg of pCR3, which confers G418 [geneticin] resistance. Cells were selected for G418 resistance in the presence of 400 µg/ml geneticin in complete growth medium. Isolated colonies arising from single cells were transferred to new plates at 14 to 21 days post-transfection (p.t.). Protein extracts prepared from these cells were subjected to immunoprecipitation (IP) and Western blot (WB) analyses to confirm viral protein expression.

### Immunoprecipitation and Western Blot (IP/WB) Analysis

Cell extracts were prepared with EBC lysis buffer (50 mM Tris, pH 8.0, 120 mM NaCl, 0.5% NP-40) supplemented with protease and phosphatase inhibitors (2 µg/ml leupeptin, 2 µg/ml E-64, 1 µg/ml aprotinin, 0.25 mM pefabloc, 1 mM sodium vanadate, 5 mM sodium fluoride, 25 mM β-glycerophosphate and 5 mM EDTA). Protein concentrations were determined by the Bradford assay using a micro protein assay (Bio-Rad Laboratories). Following initial IP experiments, the amounts of total protein in different cell lysates were adjusted to yield equivalent levels of tAg. These protein extracts were incubated with either the anti-JCV TAg monoclonal antibody PAb 962 [36] or anti-p107 antibody (C-18; Santa Cruz Biotechnology, Inc.) for one hour or with anti-PP2A-C antibody (clone ID6; Upstate Biotechnology) overnight on a rotary shaker at 4°C. Protein-antibody complexes were collected on Staph A (PANSORBIN; Calbiochem). After washing the pellets 3 times with EBC buffer, the protein complexes were disrupted by the addition of loading buffer and heating at 95°C for 4 minutes. Proteins were separated on SDS-polyacrylamide gels and transferred to nitrocellulose membranes. The membranes were incubated with a cocktail of anti-TAg monoclonal antibodies (PAb 962, 2000, 2001, 2003, 2024, 2030; [7]) to enhance detection, or with anti-p107 antibody. After exposing the membranes to BCIP/NBT (Sigma), secondary antibodies conjugated to alkaline phosphatase permitted visualization of the proteins.

### DpnI DNA Replication Assay

JCV DNA-containing vectors were digested with *Eco*RI to separate viral and plasmid DNAs. The linear viral DNA was self-ligated under dilute conditions to produce circular viral DNA. PHFG cells were seeded onto 60 mm plates and transfected 24 hours later with 100 ng of circular viral DNA using a modified DEAE-dextran protocol [37], [38]. At various time points low molecular weight DNA was extracted from the cells using the Hirt protocol [39]. This DNA was cleaved with *Dpn*I and *Eco*RI, and digestion products were electrophoresed on a 0.8% agarose gel. DNA was transferred to a nitrocellulose membrane using a Rapid Downward Transfer System and an alkaline transfer protocol (Schleicher and Schuell, Amersham). After exposure to UV light for 5 minutes to cross-link the DNA to the membrane, the DNA was hybridized with linear JCV DNA labeled with [α-^32^P] dCTP (Prime-a-Gene kit, Promega). Replication activity was determined by quantitating band intensities of linear *Dpn*I-resistant viral DNA using ImageQuant 5.2 software (Molecular Dynamics).

## Results

### JCV tAg Interacts with PP2A

**Figure 1:**
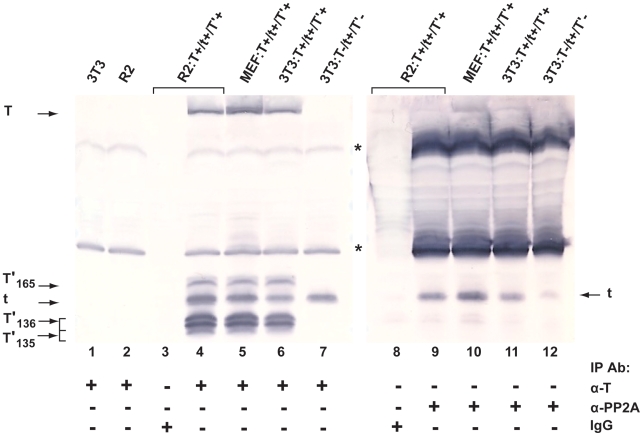
Wild type tAg interacts with cellular phosphatase PP2A in cells expressing JCV early proteins. JCV early proteins, expressed in Rat 2 (R2) or MEF cells transformed with pSR:T^+^/t^+^/T'^+^ or in G418-selected 3T3 cells transfected with pSR:T^+^/t^+^/T'^+^ (encodes all 5 JCV early proteins) or pSR:T^−^/t^+^/T'^−^ (encodes JCV tAg only) were incubated with anti-T monoclonal antibody PAb 962 (α-T; lanes 4–7) or anti-PP2A antibody (α-PP2A; lanes 9–12). The amount of total cell protein subjected to IP in lanes 9–12 was four times that employed in the corresponding samples in lanes 4–7. Immunoprecipitated proteins were separated on a 20% SDS-polyacrylamide gel, and WB analysis was performed using a cocktail of anti-T monoclonal antibodies to detect JCV early proteins either expressed in the different cell lines (lanes 4–7) or expressed and bound to PP2A (lanes 9–12). Untransfected 3T3 and Rat 2 cells were included as negative controls (no JCV T proteins are present; lanes 1, 2), and α-mouse IgG was used in the IP step with the R2:T^+^/t^+^/T'^+^ cell extract to test for non-specific binding (lanes 3, 8). The asterisks denote antibody light and heavy chains. This figure represents proteins electrophoresed on a single gel and transferred to a membrane, which was then cut in half and each half developed for different lengths of time.

The ability of polyomaviruses to induce S phase progression of a host cell is critical to the establishment of a productive infection. The early proteins of these viruses target a number of cellular factors to overcome normal cell cycle control mechanisms. The cellular phosphatase, PP2A, represents one such target as it regulates several important cell signal transduction pathways and is critical to cellular proliferation. Recently, Sariyer and co-workers [40] demonstrated an *in vitro* interaction between bacterially-produced JCV tAg fused to GST and PP2A present in extracts of U-87MG tumor cells. To demonstrate that this interaction occurs *in vivo*, lysates of Rat2, MEF and 3T3 cells expressing untagged JCV proteins were subjected to co-IP/WB analysis. A physical interaction between PP2A subunit C and JCV tAg was detected when anti-PP2A-C antibody was used for the IP step and WB was performed using anti-T protein antibodies (Fig. 1); the interaction was also detected when the order of antibodies was reversed (data not shown). These interactions between PP2A and tAg were detected in rat and mouse cells expressing all five JCV early proteins (Fig. 1, lanes 9–11), as well as in mouse cells expressing tAg only (Fig. 1, lane 12).

### Mutant JCV tAgs Interact with PP2A

**Figure 2:**
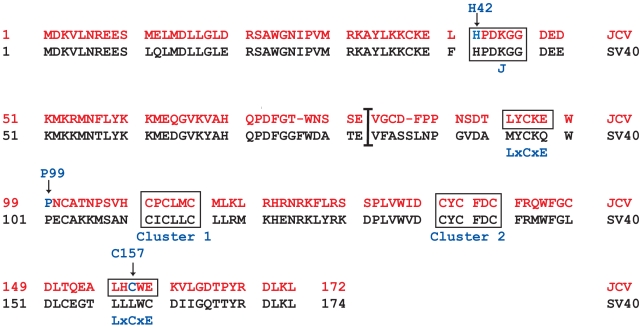
Comparison of JCV and SV40 tAg amino acid sequences. The JCV tAg sequence containing 172 amino acids is indicated in red letters, and the 174 amino acid SV40 tAg is shown in black letters. The sequences following the vertical line comprise the unique C-terminal region of each protein. Two conserved cysteine (CxCxxC) clusters (Cluster 1, 2) that contribute to SV40 tAg binding to zinc ions, and possibly to PP2A, are noted in both sequences, as are two recently recognized LxCxE motifs in JCV tAg. Three amino acid residues are highlighted with a blue letter and arrow. Histidine 42 (H42) is a conserved residue in the HPDKGG hexapeptide motif of the J domain that spans most of the N-terminal region of tAg as well as the other JCV and SV40 early proteins; H42 is required for functional interaction with Hsc70. Proline 99 is within a conserved CxxxPxC sequence that influences SV40 tAg binding to PP2A. Cysteine 157 is the central residue of the newly recognized second LxCxE motif in JCV tAg.

The unique C-terminal region of SV40 tAg contains a sequence, including two cysteines and a proline, which contributes to the tAg-PP2A interaction [23]. Alteration of any of the three residues reduces PP2A binding; substitution of alanine for the proline reduces binding to the greatest extent [23]. In addition, two motifs identified in the unique tAg sequence contain clusters of three cysteines that are involved in binding zinc ions. Mutation of any of the six cysteines in these two clusters reduces tAg stability, resulting in a half-life of 30–-60 minutes [41]; these motifs may also influence PP2A binding. The three sets of cysteine-containing sequences in the SV40 protein are conserved in the JCV tAg; CxxxPxC (amino acids 95–101) and CxCxxC (amino acids 109–114; Cluster 1 and 136–141; Cluster 2) (Fig. 2). A recent study performed with JCV tAg deletion mutants found that amino acids 82 to 124 are critical to PP2A binding in vitro [40]. Based on this information, we substituted an alanine for the conserved proline at residue 99 in the JCV tAg. This residue is also immediately adjacent to an LxCxE domain found in the JCV, but not SV40, tAg (Fig. 2). In addition, a cysteine (residue 157) present in a second LxCxE motif in the JCV tAg (residues 155–159) was changed to an alanine. Both tAg point mutants were examined for their ability to bind to PP2A and to members of the Rb family of tumor suppressor proteins.

**Figure 3:**
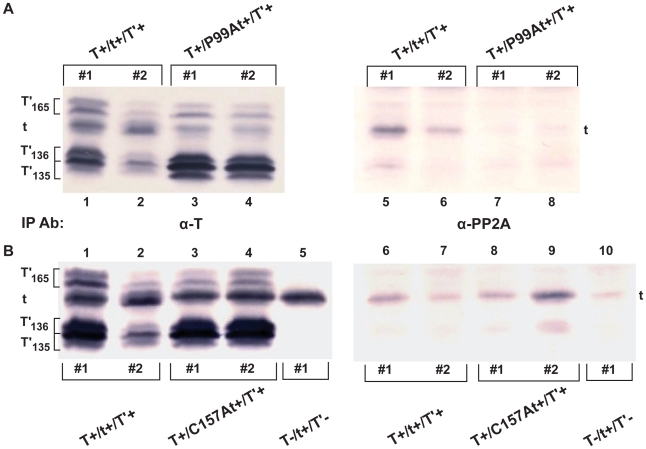
Interaction of mutant JCV tAgs with PP2A. Interactions between PP2A and wild-type tAg and (A) tAg-mutant P99A or (B) C157A were compared. 3T3 cells were stably transfected with DNA constructs expressing JCV early proteins under the control of SV40 promoter-enhancer signals. Lysates of these cells were subjected to IP with the anti-T monoclonal antibody PAb 962 (α-T) or anti-PP2A antibody (α-PP2A). The amount of total cell protein subjected to IP with anti-PP2A antibody (lanes 5–8, Panel A; lanes 6–10, Panel B) was five times that used with the anti-T antibody (lanes 1–4, Panel A; lanes 1–5, Panel B). Samples were electrophoresed on 18% SDS-polyacrylamide gels and transferred to nitrocellulose membranes. WB was performed using a cocktail of anti-T monoclonal antibodies. Results for two independently-derived cells lines (#1, #2) containing DNA constructs that either encode all 5 JCV wild type early proteins (T^+^/t^+^/T′^+^; Panel A, lanes 1,2,5,6, Panel B, lanes 1,2,6,7) or wild type TAg, T′_165_, T′_136_ and T′_135_ plus tAg mutant P99A (T^+^/P99At^+^/T′^+^; Panel A, lanes 3,4,7,8) or C157A (T^+^/C157At^+^/T′^+^; Panel B, lanes 3,4,8,9) are shown. A single 3T3 cell line expressing tAg only (T^−^/t^+^/T′^−^) was isolated and tested for PP2A binding (Panel B, lanes 5, 10). Panels A and B of this figure each represent proteins electrophoresed on a single gel and transferred to a membrane, which was then cut in half and each half developed for different lengths of time.

3T3 cells were stably transfected with DNA constructs encoding the wild type, P99A or C157A tAgs. Lysates were prepared from cells expressing all five JCV early proteins or tAg only, and total protein amounts used in co-IP experiments were adjusted to yield comparable tAg levels. These protein extracts were immunoprecipitated either with anti-T or anti-PP2A antibody and blotted with anti-T-protein antibodies (Fig. 3). Relative to PP2A-tAg interactions observed in two independently-derived cell lines expressing wild type JCV early proteins (Fig. 3A, lanes 5, 6), binding of PP2A to the mutant P99A tAg in two different lines was nearly undetectable (Fig. 3A, lanes 7, 8). The binding of mutant C157A tAg to PP2A in one cell line was similar to that of the wild type tAg (Fig. 3B, compare lane 8 with lanes 6, 7), but binding of the tAg in the second C157A line was reproducibly higher than that of wild type tAg (Fig. 3B, compare lane 9 with lanes 6, 7). Binding to PP2A was again observed in cells producing wild-type tAg in the absence of the other four JCV early proteins (Fig. 3B, lane 10). As observed in the first set of experiments (Fig. 1), the tAg-PP2A interaction in this 3T3 cell line was less robust than that observed in cells expressing all five JCV early proteins.

### JCV tAg Physically Interacts with Members of the Rb Family of Proteins

Bollag and co-workers [7] demonstrated that the LxCxE motif present in the JCV TAg and T′ proteins binds members of the Rb family of tumor suppressor proteins. While performing these co-IP/WB experiments, a 20 kDa band was observed to interact with p107 and p130. Although JCV tAg is 20 kDa, it was ruled out because an LxCxE motif had not been identified in any of the polyomavirus tAgs. Upon reexamination of the JCV sequence, we recognized two LxCxE domains in the unique C-terminal coding region of JCV tAg (Fig. 2). The first of these, residues 93–97, is common to JCV and BKV but not to SV40 or the other 3 human polyomaviruses (WUV, KIV, MCV); the second, residues 155–159, is unique to JCV tAg.

**Figure 4:**
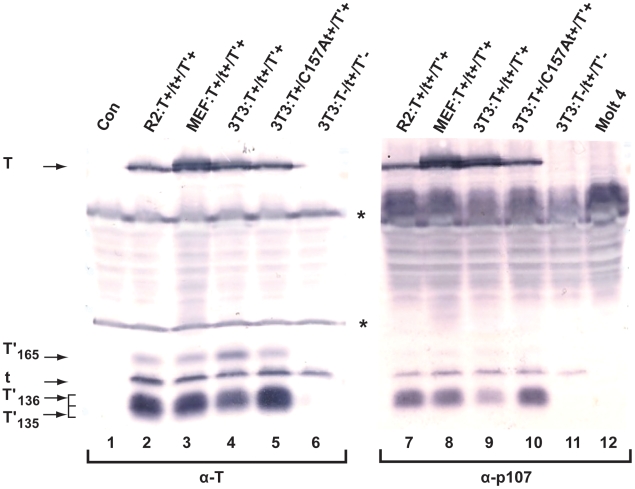
Wild type and mutant JCV tAgs bind the Rb protein p107. JCV early proteins expressed in Rat 2 (R2) or MEF cells transformed with pSR:T^+^/t^+^/T'^+^ (encodes all 5 JCV early proteins) or in G418-selected 3T3 cells stably transfected with pSR:T^+^/t^+^/T'^+^, pSR:T^−^/t^+^/T'^−^ (encodes tAg only) or pSR:T^+^/C157At^+^/T'^+^ (encodes tAg mutated at residue 157 and the other 4 JCV early proteins) were incubated with anti-T monoclonal antibody PAb 962 (α-T; lanes 2–6) or anti-p107 antibody (α-p107; lanes 7–11). Negative controls included EBC lysis buffer immunoprecipitated with anti-T monoclonal antibody PAb 962 (Con, lane 1) and Molt 4 extract with anti-p107 antibody (Molt 4, lane 12). The amount of total cell protein subjected to IP in lanes 7–11 was 10 times that employed in the corresponding samples in lanes 2–6. Proteins were separated on an 18% SDS-polyacrylamide gel and WB analysis was performed using a cocktail of anti-T monoclonal antibodies. The asterisks denote antibody light and heavy chains. This figure represents proteins electrophoresed on a single gel and transferred to a membrane, which was then cut in half and each half developed for different lengths of time.

To determine whether JCV tAg interacts with the Rb family of proteins, we performed co-IP/WB upon lysates from cells expressing all five JCV early proteins or tAg alone, and with total protein amounts adjusted to comparable levels of tAg expression in each cell line. Lysates were incubated with Molt 4 extracts overnight to compensate for the low levels of expression of Rb proteins in the rat and mouse cells [7], [8], immunoprecipitated with anti- p107 antibody and immunoblotted with a cocktail of antibodies that recognize the JCV early tumor proteins. All five JCV early proteins bound p107. The interaction between T′_165_ and p107 is difficult to detect, not only because T′_165_ binding is inefficient [7], but also because it is expressed at low levels relative to the other early viral proteins. Wild type JCV tAg present in both rat and mouse cells expressing the intact JCV early region was found to interact comparably with p107 (Fig. 4, lanes 7–9), while the binding of tAg to p107 without co-expression of the other four early proteins was only slightly decreased (Fig. 4, lane 11). The C157A tAg which carries a mutation that disrupts one LxCxE domain, also interacted with p107 (Fig. 4, lane 10). Comparable binding of the wild type and mutant tAgs to p130 was also detected (data not shown). As noted with the other JCV early proteins [7], [8], binding of wild type tAg to pRB was difficult to detect, most likely because levels of this tumor suppressor protein are low in these cells (data not shown; [7]).

### JCV tAg Contributes to Viral DNA Replication in Permissive Cells

**Figure 5:**
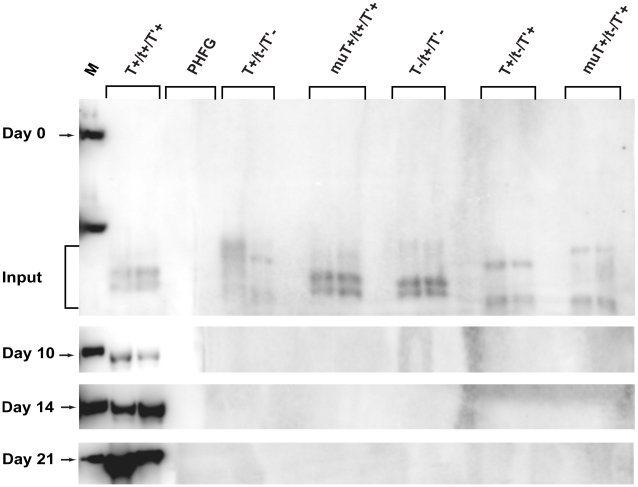
Replication efficiencies of DNA constructs expressing combinations of JCV early proteins. PHFG cells in 60 mm plates were transfected in duplicate with 400 ng of wild type (T^+^/t^+^/T'^+^) or mutant (T^+^/t^−^/T'^−^, muT^+^/t^+^/T'^+^, T^−^/t^+^/T'^−^, T^+^/t^−^/T'^+^, or muT^+^/t^−^/T'^+^) JCV genomes. At each time point, viral DNA was extracted by the Hirt procedure [39], purified, digested with *Dpn*I and *Eco*RI, electrophoresed on a 0.8% agarose gel, transferred to nitrocellulose membranes and hybridized to a [P^32^]dCTP-labeled JCV DNA probe. Bands were visualized with a Typhoon phosphorimager and quantitated with ImageQuant software. The marker (M) shown in the first lane of each blot is 1 ng of linear JCV DNA (5130 bp). The duplicate, independent samples representing each DNA construct were analyzed, and the position of *Dpn*1-resistent replicating genomes on the Southern blots is denoted by an arrow at days 10, 14 and 21 p.t. *Dpn*1- and *Eco*RI-sensitive input DNAs are noted at the day 0 time point. Hirt extracts of uninfected PHFG cells (PHFG) were subjected to the same Dpn1 assay.

SV40 TAg is recognized as the primary DNA replication protein. It is the only viral protein required to initiate viral DNA replication in a cell-free system [42], and in cultured cells, SV40 genomes encoding TAg without tAg are viable, although virus titers may be reduced relative to parental SV40 [43]. JCV TAg is also necessary and sufficient to promote viral DNA replication in a cell-free system [44]. However, *in vivo*, the JCV T′ proteins contribute to replication potential, and the contributions of tAg are unknown [45]. To examine the individual roles of the JCV early proteins in viral DNA replication, the intact wild-type JCV genome (JR:T^+^/t^+^/T′^+^), a TAg only mutant (JR:T^+^/t^−^/T′^−^), a TAg deletion mutant (JR:muT^+^/t^+^/T′^+^), a tAg only mutant, (JR:T^−^/t^+^/T′^−^), a tAg null mutant (JR:T^+^/t^−^/T′^+^), and a T′ only mutant (JR:muT^+^/t^−^/T′^+^) were each transfected into two plates of PHFG cells (Fig. 5). Low-molecular DNA was extracted from the cells at several times p.t., and DNA replication was measured using a DpnI assay [46]. At day 0, equivalent amounts of input DNA were detected for each sample; at all subsequent time points, days 10, 14, and 21 p.t., increasing levels of the replicated wild type JCV genome were detected. No replication of any DNA construct lacking either TAg or tAg was observed *in vivo* at any time point.

**Figure 6:**
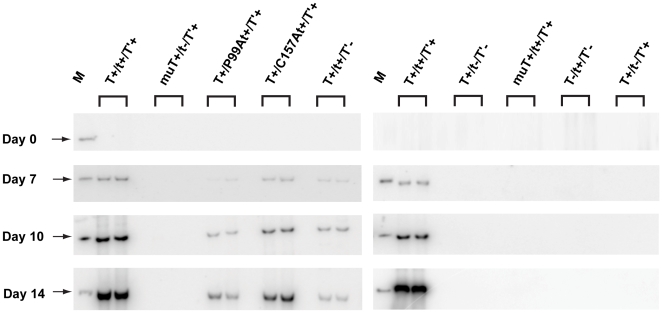
Mutation of tAg residues proline 99 and cysteine 157 reduce JCV DNA replication. PHFG cells in 60 mm plates were transfected in duplicate with 400 ng of wild type (T^+^/t^+^/T'^+^) or 8 different mutant (muT^+^/t^−^/T'^+^, T^+^/P99At^+^/T'^+^, T^+^/C157At^+^/T'^+^, T^+^/t^+^/T'^−^, T^+^/t^−^/T'^−^, muT^+^/t^+^/T'^+^, T^−^/t^+^/T'^−^, T^+^/t^−^/T'^+^) JCV genomes. Duplicate, independent samples representing each DNA construct were extracted by the method of Hirt [39] on days 0, 7, 10 and 14 p.t. and analyzed using the Dpn1 assay as described in the legend to Figure 5. The marker (M) shown in the first lane of each blot is 1 ng of linear JCV DNA (5130 bp), and the position of *Dpn*1-resistent replicating genomes is denoted by an arrow at days 7, 10 and 14 p.t.

To further examine tAg's role in viral DNA replication, the Dpn1 assay was repeated with the same DNA constructs used in Figure 5 plus the two tAg substitution mutants (JR:T^+^/P99At^+^/T′^+^ and JR:T+/C157At^+^/T′^+^) and a T′ null mutant (JR:T^+^/t^+^/T′^−^) (Fig. 6). At day 0, only input DNA was detected (data not shown), but at later time points, increasing levels of *Dpn*1-resistant replicated JCV DNA were observed with the two tAg point mutants and the T′ deletion mutant; the other DNA constructs exhibited the same replication behavior observed in the previous experiment (Fig. 5). Genomes containing the P99A and C157A tAg mutations exhibited reduced replication potential relative to wild type JCV DNA (3–4% and 20–29%, respectively, depending on the time point). As reported earlier [45], JR:T^+^/t^+^/T′^−^ DNA replication was reduced more than 10-fold (6–8% of wild type). DNA replication of the tAg null mutant was not detected at any time point, confirming the results presented in Figure 5 indicating that JCV tAg has a significant influence upon this critical activity.

### Expression of JCV tAg in *trans* does not Relieve the Replication Defect of JR:T^+^/t^−^/T′^+^

**Figure 7:**
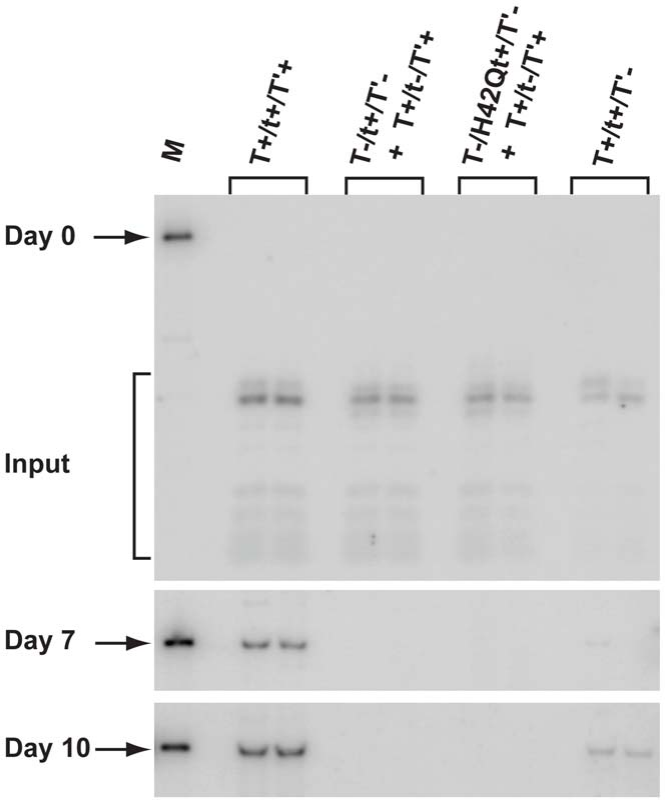
JCV tAg fails to complement replication of a tAg-deficient JCV genome in *trans*. PHFG cells in 60 mm plates were co-transfected in duplicate with 400 ng of a tAg-deficient (T^+^/t^−^/T'^+^) JCV genome and 400 ng of a JCV DNA construct expressing either wild type tAg (T^−^/t^+^/T′^−^) or a J domain mutant tAg (T^−^/H42Qt^+^/T′^−^) under the control of the JCV promoter-enhancer. Cells were also transfected with 400 ng of either a T′-deficient (T^+^/t^+^/T'^−^) JCV genome or a positive replication control (T^+^/t^+^/T'^+^). Duplicate, independent samples representing each DNA construct were extracted by the method of Hirt [39] on days 0, 7, and 10 p.t. and analyzed using the Dpn1 assay as described in the legend to Figure 5. The marker shown in the first lane of each blot is 1 ng of linear JCV DNA (5130 bp), and the position of *Dpn*1-resistent replicating genomes is denoted by an arrow at days 7 and 10 p.t. *Dpn*1- and *Eco*RI-sensitive input DNAs are noted at the day 0 time point.

An SV40 H42Q mutant altered at a critical residue of the shared J domain of the three early proteins is replication-defective [16]. We created an H42Q mutant of JCV tAg to see if this alteration impacted tAg's ability to support DNA replication. Because the mutation affected the other four JCV early proteins as well, we introduced the H42Q mutation into a construct that only expresses tAg to generate JR:T^−^/H42Qt^+^/T′^−^. This DNA, or a DNA expressing wild type tAg only, was co-transfected into cells with a construct expressing wild type TAg and the three T′ proteins, but not tAg (JR:T^+^/t^−^/T′^+^), to determine if tAg expression complemented replication of the latter DNA in *trans*. *Dpn*1-resistent DNA was not detected using either combination of co-transfected DNAs at any time point, although replication was observed with the wild type control construct (JR:T^+^/t^+^/T′^+^) and to a lesser extent with the construct lacking the T′ proteins (JR:T^+^/t^+^/T′^−^) (Fig. 7). The absence of detectable complementation with the wild type tAg precludes a determination of whether the H42Q mutation in tAg impacts this protein's replication function.

### Elevated Expression of JCV TAg and T′ Proteins Promotes DNA Replication in the Absence of tAg

**Figure 8:**
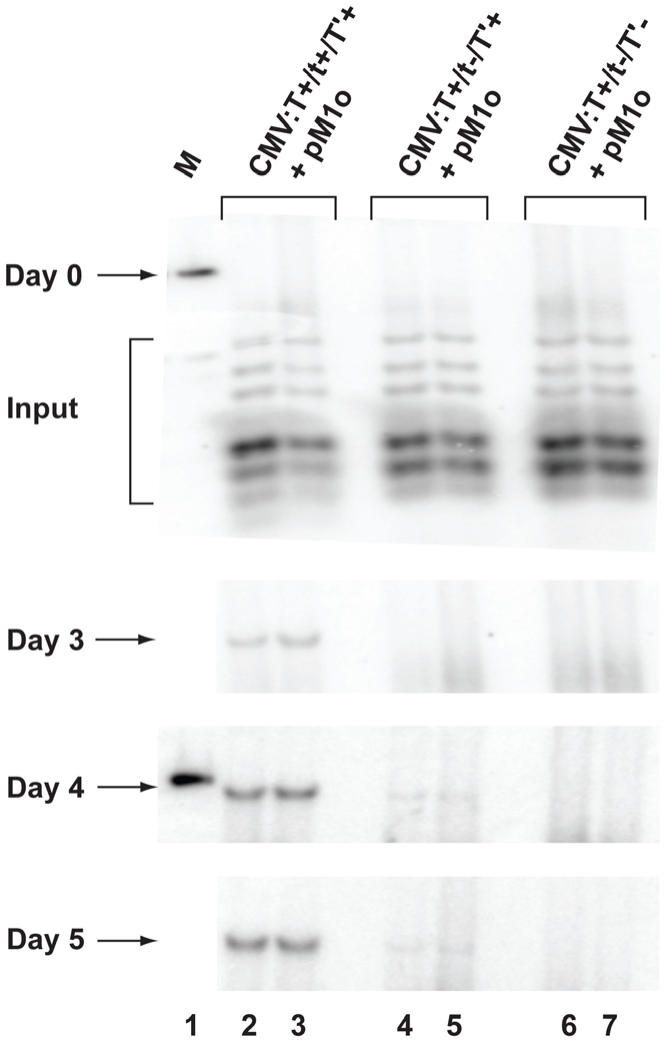
JCV early genes expressed from a CMV promoter induce replication of a JCV origin plasmid. PHFG cells in 60 mm plates were co-transfected in duplicate with 400 ng of the JCV replication origin plasmid, pM1o, and 400 ng of one of the following expression vectors that encode JCV early genes under the control of the CMV promoter: pCMV:T^+^/t^+^/T′^+^ (lanes 2, 3; all five tumor proteins expressed), pCMV:T^+^/t^−^/T′^+^ (lanes 4, 5; TAg and 3 T′ proteins expressed) or pCMV:T^+^/t^−^/T′^−^ (lanes 6, 7; TAg only expressed). The ability of the proteins produced by the second plasmid to drive replication of the origin plasmid in *trans* was tested. Duplicate, independent samples representing each DNA construct were extracted by the method of Hirt [39] on days 0, 3, 4 and 5 p.t. and analyzed using the Dpn1 assay as described in the legend to Figure 5. The marker (M) shown in lane 1 of each blot is 1 ng of linear pM1o (∼2400 bp), and the position of *Dpn*1-resistent replicating genomes is denoted by an arrow at days 3–5 p.t. *Dpn*1- and *Eco*RI-sensitive input DNAs are noted at the day 0 time point.

The levels of viral proteins expressed from the JCV transcriptional signals are low relative to the levels induced by the SV40 signals [35], [47]. We hypothesized that if JCV early protein expression was increased, viral DNA replication might be elevated to detectable levels for some replication-defective JCV constructs, including the tAg deletion mutant. Although the SR:T^+^/t^−^/T′^+^ construct might be considered a logical choice to test this prediction, the JCV early proteins in this chimeric DNA do not productively interact with the SV40 origin sequences to mediate viral DNA replication [38]. To overcome this obstacle, PHFG cells were co-transfected with two DNAs, the first was a construct containing the JCV origin of replication (pM1o; [38]) and the second was one of three constructs containing wild type (T^+^/t^+^/T′^+^) or mutant (T^+^/t^−^/T′^+^ or T^+^/t^−^/T′^−^) JCV early coding regions under the control of a CMV promoter. This DpnI assay differed from those described above in at least three ways: (i) the CMV promoter is more active than the JCV transcriptional signals and the T proteins are expressed at higher levels, (ii) the constructs contain the JCV early coding region linked to plasmid sequences rather than the intact JCV genome and (iii) the JCV proteins and replication origin are not resident on the same DNA molecule. Following co-transfection of PHFG cells, replicated viral DNA was isolated at days 0, 3, 4, and 5 p.t. Replication of the origin plasmid pM1o was detected at days 3, 4 and 5 p.t. in the presence of pCMV:T^+^/t^+^/T′^+^ (Fig. 8). At days 4 and 5 p.t., the small t null construct, pCMV:T^+^/t^−^/T′^+^, mediated replication of the pM1o vector at less than 10% the level observed in the presence of all 5 JCV early proteins. No replication was detected at any time point in the presence of the construct expressing TAg only (pCMV:T^+^/t^−^/T′^−^), suggesting that at least some contributions of the tAg and the T′ proteins to DNA replication are unique.

## Discussion

A substantial number of biological differences have been documented between the primate polyomaviruses JCV and SV40, yet the sequence and the organization of the two viral genomes are remarkably similar [2]. Early studies employing chimeric viruses indicated the transcriptional control regions of each virus, including their viral promoter and enhancer elements, have a significant impact on the individual transforming and lytic behaviors of JCV vs. SV40 [35], [38], [48], [49]. These sequences have diverged to the greatest extent with only 44% sequence identity between JCV and SV40. Experiments with the hybrid viral genomes also revealed the early coding regions to be sources of biological variation [35], [48], [50]. Although the N-terminal region of the tAgs share 82% sequence identity, the unique C-terminal portions, with only 54% identity, represent the sequences with the least amount of similarity among the early proteins; these sequences were the primary focus of the current study.

Very little work has been conducted with the JCV tAg, although numerous studies with its SV40 counterpart suggest interesting initial experiments to perform. The binding of the unique region of the SV40 tAg to PP2A provides the mechanism by which tAg induces most observed alterations to the behavior and morphology of cells [22], [31], [51]–[54]. The only other interaction predicted for SV40 tAg occurs with the molecular chaperone Hsc70 via its J domain located within the N-terminal 82 amino acid sequences shared with TAg and 17kT. JCV tAg may bind to these and additional cellular and viral proteins. Purified GST-JCV tAg prepared in bacteria binds PP2A *in vitro* [40], and JCV tAg expressed in mammalian cells binds PP2A *in vivo* (current study). Many of the outcomes reported to result from the SV40 tAg-PP2A interaction, including those involving the activation of the PI3K and MAPK pathways, are likely to occur following contact between JCV tAg and PP2A. The JCV tAg has been shown to interfere with the phosphatase activity of PP2A in studies involving a second known binding partner of tAg, the JCV agnoprotein [40].

We were surprised to identify two LxCxE motifs that had been overlooked in the unique region of the JCV tAg; one of these sites is also found in BKV but neither site resides in the corresponding SV40, WUV, KIV or MCV polyomavirus proteins. As predicted, JCV tAg binds members of the retinoblastoma protein family. These interactions are relevant to the potential contributions tAg may make to JCV oncogenic activities. While the tAg-PP2A interaction may activate signaling pathways critical to cell growth and survival, effects on Rb proteins may also influence these processes via cell cycle regulation. The LxCxE motif and the tripeptide HDP of the J domain of the polyomavirus tumor proteins make direct contacts with the Rb proteins and Hsc70, respectively; together they effect the release of members of the E2F family of transcription factors from their Rb partners to promote cell cycle progression [reviewed in 55].

There is less certainty about the identity of tAg amino acids that are in direct contact with PP2A. Mutations introduced into the two CxCxxC clusters in the C terminus of SV40 tAg yield an unstable protein and disrupt zinc binding [23]. Under these conditions, the role of these two clusters in PP2A binding could not be determined. Mutations to the two cysteines and one proline of the CxxxPxC motif located upstream of the CxCxxC clusters did reduce PP2A binding significantly. Additional studies that relied upon peptide inhibition of the tAg-PP2A interaction without destabilizing tAg suggested that the CxCxxC clusters might indeed influence tAg binding to the A subunit [23], [25]. Two recent X-ray crystallographic studies indicate the CxCxxC clusters and the CxxxPxC sequence in the C terminus of SV40 tAg and the HDPKGG sequence of the N-terminal J domain contribute to tAg binding to the scaffolding A and catalytic C subunits of PP2A [53], [54].

To begin to identify JCV tAg residues critical to PP2A and Rb interactions and to DNA replication activity, we introduced mutations into the conserved CxxxPxC sequence and the unique LxCxE motif within the C-terminus of JCV tAg. Using a co-immunoprecipitation approach, we confirmed our prediction that the P99A mutant tAg would exhibit a loss of PP2A binding activity. This proline is conserved in the tAgs of primate polyomaviruses as well as of mouse polyoma virus.

We created the C157A tAg mutant to determine if the conserved cysteine in the newly recognized LxCxE domain of JCV tAg was required for Rb binding. However, once made, we also tested whether the mutant displayed altered PP2A binding relative to wild type tAg. In one cell line, we found that the C157A tAg bound PP2A with wild type efficiency. We consistently observed; however, that the same mutant tAg expressed in a second, independently derived cell line bound PP2A even better than wild type tAg. A preliminary comparison of the two cell lines expressing the C157A tAg indicates that the line in which more efficient PP2A binding occurred had a shorter doubling time and more transformed morphology. This observation will require additional investigation to determine if enhanced PP2A binding is associated with a transformed phenotype.

It should be noted that to detect PP2A binding in this study, we employed antibodies that recognized the catalytic subunit of PP2A. PP2A is found abundantly as either a holoenzyme consisting of the AC core plus a regulatory B subunit, or as the AC core alone. High levels of the A and C subunits are not found as free proteins in cells [56], and SV40 tAg does not bind detectably to purified free C subunit [57]. Given this information, our data suggest JCV tAg binds the scaffold subunit (A) of the AC core of PP2A in rat and mouse fibroblasts and not directly to the C subunit.

As noted above, the C157A tAg mutant was created to see if alteration of the second LxCxE motif would affect Rb binding. In addition, the CxxxPxC motif containing the P99A mutation overlaps the first LxCxE domain in the JCV protein. Neither mutation had a detectable affect on the ability of tAg to bind p107 (Fig. 4) or p130 (data not shown). It is likely that both LxCxE motifs contribute to Rb binding, perhaps redundantly, and that to abolish binding activity, double mutants will have to be created and tested. It is also possible that neither LxCxE motif is required for Rb binding, as a number of cellular proteins lacking this sequence do functionally interact with pRB, p107 and p130 [58]. Furthermore, sequences in addition to the LxCxE motif contribute to the ability of some viral oncoproteins to bind Rb proteins [59], [60].

SV40 TAg has long been recognized at the key replication protein of this virus, and *in vivo* and *in vitro* studies have confirmed this role for JCV TAg as well. Regulation of SV40 DNA replication involves the incompletely understood coordination of TAg phosphorylation and dephosphorylation. Phosphorylation of serines 120 and 123 negatively regulates TAg functions, whereas phosphorylation of threonine 124 is required for TAg-mediated replication [reviewed in 20], [21]. Modifications to the corresponding residues in JCV TAg, serines 121 and 124 and threonine 125, have been proposed, and threonine 125 has been demonstrated to be essential to replication activity [61], [62]. A cell-free DNA replication system utilizing SV40 TAg indicated that addition of PP2A enhances DNA replication and that SV40 tAg inhibits this effect by blocking dephosphorylation of serines 120 and 123 [63], [64]. However, SV40 tAg appears to stimulate SV40 replication to a limited extent *in vivo*; SV40 tAg deletion mutants produce slightly lower numbers of virions per infected cell [43] and addition of tAg protein to cells infected with SV40 tAg-defective genomes stimulates DNA replication [20]. Recently, tAg was shown to have a significant effect on DNA replication of the human polyomavirus MCV [65]. In the absence of tAg, TAg-mediated replication is reduced nearly 10-fold in 293 cells. Nesper et al. [44] developed a cell-free replication system to demonstrate that TAg is the only JCV protein required to mediate *in vitro* replication of a plasmid containing the JCV origin of replication. Using an *in vivo* approach with permissive PHFG cells, it was shown that JCV TAg is required for DNA replication, and JCV T′ proteins contribute to replication efficiency [45]. Further, a truncated JCV tAg mutant, encoding amino acids 1–89, replicated with similar efficiency to wild type JCV at days 7 and 14 p.t., but significant inhibition was observed at a later time point [40]. The authors speculated that reduced replication resulted from the inability of the mutant tAg to inhibit PP2A, thereby leading to dephosphorylation of the late JCV agnoprotein and inefficient DNA replication. In the current study we found that a JCV genome missing only tAg failed to replicate to detectable levels in PHFG cells. By modifying the replication assay and increasing expression of the JCV early proteins under the control of more potent CMV transcriptional signals, low levels of JCV DNA replication (∼10% of wild type; Fig. 8) were observed; a construct lacking tAg and the three T′ proteins, however, still failed to replicate under these conditions. The inability of the CMV:T^+^/t^−^/T′^−^ construct to drive replication of the JCV origin plasmid suggests that not all contributions of the tAg and the T′ proteins to DNA replication are redundant. These data indicate that JCV tAg, unlike its SV40 counterpart but similar to MCV tAg, makes a significant contribution to viral DNA replication activity, and furthermore, that its effect on agnoprotein phosphorylation status does not completely explain its role in replication, since agnoprotein was not present in our assay. We speculate that interactions of tAg with PP2A and Rb proteins alter JCV TAg phosphorylation status and promote cell cycle progression, leading to conditions more favorable to viral DNA replication.

We also tested whether the H42Q, P99A and C157A mutations in JCV tAg had any effect on viral DNA replication. Because the latter two mutations reside in the unique coding sequences of tAg, this protein could be altered without affecting the other four early proteins. However, the H42Q mutation affects the shared N terminus of all five early proteins. To determine if the H42Q mutation affected DNA replication behavior, we co-transfected the JR:T^+^/t^−^/T′^+^ genome into PHFG cells with either the wild type or H42Q mutant tAg construct. We predicted this approach would allow us to determine whether H42Q tAg complemented the tAg-defective genome (i.e. JR:T^+^/t^−^/T′^+^). While no replication was observed in this case, the control which employed the wild type tAg also failed to complement the tAg-defective DNA, and no conclusion could be reached regarding the influence of this J domain residue upon JCV DNA replication. This experiment does; however, provide new information indicating that expression of tAg in *trans* fails to correct the replication defect of a tAg-deficient JCV genome. It is possible that the levels of tAg produced from the JCV transcriptional signals are insufficient to permit detection of a complementing activity. It should be noted that a similar mutation to the J domain of the MCV tAg did not alter DNA replication behavior, while the same mutation in the MCV TAg markedly reduced this activity [65].

We demonstrated that the P99A mutant, and to a lesser extent the C157A mutant, exhibits a significant reduction in DNA replication relative to that observed with wild-type JCV. Thus, proline 99 and cysteine 157 are likely to either affect tAg's ability to cooperate with the other T proteins or with cellular proteins that influence DNA replication potential and/or cell proliferation and survival. We have shown that the P99A mutation alters one such interaction, i.e. binding to PP2A. Significantly, Kwun and co-workers [65] reported that a mutation to a CxCxxC motif in the MCV tAg, abrogated the ability of this protein to stimulate DNA replication. The authors suggested that loss of binding to PP2A by the MCV mutant tAg affected the phosphatase's ability to modify cellular or viral targets that impact replication either directly or indirectly.

JCV tAg possesses unique features among polyomavirus small t proteins. Unlike SV40 tAg, it plays a significant role in the replication of viral DNA *in vivo*. SV40 tAg has been found to interact with PP2A, and it is predicted to bind Hsc70. In addition to these two cellular proteins, JCV tAg binds to the viral agnoprotein and to the Rb proteins, pRB, p107 and p130, most likely via two unique LxCxE motifs. Cooperation between the LxCxE and J domains of JCV tAg are expected to influence cell cycle progression. The study of JCV tAg has only recently been pursued, and it is expected that a greater understanding of this viral tumor protein will provide new insights into the pathogenic and oncogenic potentials of this human virus.
